# Dynamic Panel Estimate–Based Health Surveillance of SARS-CoV-2 Infection Rates to Inform Public Health Policy: Model Development and Validation

**DOI:** 10.2196/20924

**Published:** 2020-09-22

**Authors:** James Francis Oehmke, Theresa B Oehmke, Lauren Nadya Singh, Lori Ann Post

**Affiliations:** 1 Department of Emergency Medicine Feinberg School of Medicine Northwestern University Chicago, IL United States; 2 Department of Civil Engineering University of California at Berkeley Berkeley, CA United States

**Keywords:** COVID-19, models, surveillance, COVID-19 surveillance system, dynamic panel data, infectious disease modeling, reopening America, COVID-19 guidelines, COVID-19 health policy

## Abstract

**Background:**

SARS-CoV-2, the novel coronavirus that causes COVID-19, is a global pandemic with higher mortality and morbidity than any other virus in the last 100 years. Without public health surveillance, policy makers cannot know where and how the disease is accelerating, decelerating, and shifting. Unfortunately, existing models of COVID-19 contagion rely on parameters such as the basic reproduction number and use static statistical methods that do not capture all the relevant dynamics needed for surveillance. Existing surveillance methods use data that are subject to significant measurement error and other contaminants.

**Objective:**

The aim of this study is to provide a proof of concept of the creation of surveillance metrics that correct for measurement error and data contamination to determine when it is safe to ease pandemic restrictions. We applied state-of-the-art statistical modeling to existing internet data to derive the best available estimates of the state-level dynamics of COVID-19 infection in the United States.

**Methods:**

Dynamic panel data (DPD) models were estimated with the Arellano-Bond estimator using the generalized method of moments. This statistical technique enables control of various deficiencies in a data set. The validity of the model and statistical technique was tested.

**Results:**

A Wald chi-square test of the explanatory power of the statistical approach indicated that it is valid (χ^2^_10_=1489.84, *P*<.001), and a Sargan chi-square test indicated that the model identification is valid (χ^2^_946_=935.52, *P*=.59). The 7-day persistence rate for the week of June 27 to July 3 was 0.5188 (*P*<.001), meaning that every 10,000 new cases in the prior week were associated with 5188 cases 7 days later. For the week of July 4 to 10, the 7-day persistence rate increased by 0.2691 (*P*=.003), indicating that every 10,000 new cases in the prior week were associated with 7879 new cases 7 days later. Applied to the reported number of cases, these results indicate an increase of almost 100 additional new cases per day per state for the week of July 4-10. This signifies an increase in the reproduction parameter in the contagion models and corroborates the hypothesis that economic reopening without applying best public health practices is associated with a resurgence of the pandemic.

**Conclusions:**

DPD models successfully correct for measurement error and data contamination and are useful to derive surveillance metrics. The opening of America involves two certainties: the country will be COVID-19–free only when there is an effective vaccine, and the “social” end of the pandemic will occur before the “medical” end. Therefore, improved surveillance metrics are needed to inform leaders of how to open sections of the United States more safely. DPD models can inform this reopening in combination with the extraction of COVID-19 data from existing websites.

## Introduction

### Background

The SARS-CoV-2 pandemic is unprecedented [1,2], with high mortality and morbidity of the virus due to its rapid spread worldwide [3,4]. Without an effective vaccine [5-7], countries are at risk for continued spread [8]. Without good health surveillance, public health leaders are unaware of where and how the disease is spreading. Effective surveillance can inform the safe reopening of economies [9-22] by geographical region [23]. To that end, we submit this proof of concept of the creation of surveillance metrics that correct for measurement error and data contamination. This study applies state-of-the-art statistical modeling to existing data mined from the internet to derive the best available estimates of the state-level dynamics of COVID-19 infection to determine if the sustained decline in SARS-CoV-2 infection that is necessary to reopen is occurring or, conversely, if reopening without applying best public health practices is resulting in a resurgence of SARS-CoV-2.

Public health surveillance is defined as the “ongoing systematic collection, analyses, and interpretation of outcome-specific data for use in the planning, implementation and evaluation of public health practice [18].” Unfortunately, existing surveillance methods suffer from undercounts, bias, and error, and they mostly include more severe cases [24-32]. Research has confirmed that best practices for containment of the COVID-19 pandemic include closing borders between countries [33,34], extreme quarantine measures [35-37], social isolation at home [38], social distancing [39], hand hygiene [40-42], crowd control [43], and wearing a mask in public [44,45]; however, health surveillance must inform where and when to employ these best practices. Due to delays in reporting of new cases, deaths, and testing [46-48], these decisions are made based on partial evidence. Existing models of COVID-19 contagion rely on parameters such as the basic reproduction number (R_0_), which are difficult to measure in real time, and they use static statistical methods that do not capture all of the relevant dynamics [49], such as varying specificity and sensitivity of diagnostic testing or asymptomatic individuals who are never tested and are unwittingly carrying SARS-CoV-2 [25,50]. The epidemiological definition of R_0_ is the average number of people who contract a disease from a contagious person. It applies specifically to a population of people who were previously free of infection and were not vaccinated [51]. Existing surveillance systems use data that are subject to significant measurement error and other contaminants [52,53]. Moreover, timely information is needed to improve statistical methods that extract information from data sets posted on websites [54-56].

The conventional approach to modeling the spread of diseases such as COVID-19 is to posit an underlying contagion model [57] and then to seek accurate direct measurement of the model parameters, such as reproduction rates or other parameters; these measurements are sometimes inferred through deaths, hospitalizations, and caseloads [58], and they often involve labor-intensive methods that rely on contact tracing to determine the spread of the disease among a sample population [54,59-61]. For viral epidemics with an incubation period of up to 14 days [62], weeks if not months are required to generate accurate parameter estimates, even for simple contagion models. For example, early estimates of COVID-19 were estimated using methods developed by Lipsitch [63] applied to data from contact tracing in Wuhan and Italy; however, the statistical properties were weak [64-70]. For example, Zhao [65] estimated the serial interval distribution and R_0_ based on only six pairs of cases [71]. These models also rely on underlying assumptions about immunity, common propensity for infection, and well-mixed populations, among others. Improvements in these models typically focus on relaxing these assumptions, such as disaggregating the population by geography and modeling within-geography and cross-geography personal interactions [3]. Martcheva [76] provides an excellent dynamic analysis of a wide variety of contagion models and their possible dynamics [72-77]. Unfortunately, they provide limited options for the statistical inference of parameter values from actual data [76]. The objective of this study is to derive surveillance metrics using methods that control for data limitations and contamination.

## Methods

### Model Development

In contrast to previous studies, we used an empirical approach that focuses on statistical modelling of widely available empirical data, such as the number of confirmed cases or the number of tests, which can inform estimates of the current values of critical parameters such as the infection rate or reproduction rate. We explicitly recognized that the data generating process for the reported data contains an underlying contagion component; a politico-economic component, such as availability of accurate test kits; a social component, such as how strongly people adhere to social distancing measures, mask requirements, and shelter-in-place policies; and a sometimes inaccurate data reporting process that may obscure the underlying contagion process. Therefore, we sought to develop a statistical approach that can provide meaningful information despite the complex and sometimes obfuscating data generation process. Our approach is consistent with the principles of evidence-based medicine, including controlling for complex pathways that may include socioeconomic factors such as mediating variables and policy recommendations, and “based on the best available knowledge, derived from diverse sources and methods [5].”

There are two primary advantages to this empirical approach. First, we can apply the empirical model relatively quickly to a short data set. This advantage stems from the panel nature of the model. We used US states as the cross-sectional variable; therefore, one week of data from 52 states and territories (including Puerto Rico and the District of Columbia) provides a reasonable sample size. In addition to enabling parameter estimation early in a pandemic, using this property, we tested to see if a shift had occurred in the infection or reproduction rates of the contagion process in the past week (ie, whether there is statistical evidence that reopening is associated with an acceleration in the number of cases).

The second advantage of our approach is that it directly measures and informs policy-relevant variables. For example, the White House issued guidance on reopening the US economy that depends on a decrease in the documented number of cases and in the proportion of positive test results over a 14-day period, among other criteria and considerations [23,78-83]. As noted above, the number and proportion of positive test results are the outcomes of a data generating process that includes not only the underlying contagion process but a multitude of mediating factors as well as idiosyncrasies of the data collection and a delayed reporting process. We specifically modeled the number of positive test results in our empirical model, which provides evidence of direct use in policy dialogue.

Herein, we proceed with a brief discussion of the contagion models that informed our selection of an empirical model. We describe the basic dynamic panel data (DPD) approach and its advantages for analyzing the current pandemic. We obtained results that validate the model specification, which is a necessary and important step in the development of a surveillance system [9-11,14,15,18,20]. We then used the validated model to interrogate our research question: is reopening associated with increased infection transmission and a re-emergence of the pandemic? We approached this research question by statistically testing whether R-type contagion parameters and, specifically, the daily and weekly persistence increased during the weeks of June 27-July 3 and July 4-10, 2020.

### Representing Contagion as a DPD Model

Transmission models are typically population-based differential equations of the form *dY/dt = f*(*Y*,*X*), where *Y* is a vector of a population or subpopulation characteristic of interest, such as the number of exposed or infected individuals; *X* is a vector of mediating factors (often omitted); and *f* is a transition function. For empirical purposes, we will use difference equations because the data come in discrete time periods, specifically days. For example, the sizes of the susceptible, infected, and recovered populations in the susceptible-infected-recovered (SIR) model in difference equation form are:

*■(S_it- S_(it-1) = - (βS_(it-1) I_(it-1))⁄N_(it-1) @I_it- I_(it-1) = (βS_(it-1) I_(it-1))⁄N_(it-1) - 〖(γ〗_R+γ_D)I_(it-1)@■(R_it- R_(it-1)= γ_R I_(it-1) @D_it- D_(it-1)= γ_D I_(it-1) ))***(1)**

where *S*, *I*, and *R* are the sizes of the susceptible, infected, and recovered populations, respectively; *D* is the number of deaths due to SARS-CoV-2; *N* is the size of the total population (*S* + *I* + *R* + *D*); and the subscripts denote the time period. The first line represents the change in the susceptible population, which decreases when a susceptible individual becomes infected. This occurs when the susceptible individual interacts with another individual who is infected, in which case the virus is transmitted to the susceptible individual with probability *I*/*N*. The number of infected individuals increases by the number of newly infected individuals and decreases by the number of previously infected individuals who either recovered or died. The *γ* parameters are the probability of recovering or dying. *β* and the *γ* are the unknown parameters of the model. Calibration of contagion models requires estimation of the true parameter values.

The availability of state-level data suggests that Equation 1 can be rewritten in panel regression form as

*■(S_it = γ_Si+S_(it-1)- (βS_(it-1) I_(it-1))⁄N_(it-1) +ε_Sit @I_it = γ_Ii+((1+ βS_(it-1))⁄N_(it-1) - 〖(γ〗_R+γ_D))I_(it-1)+ε_Iit@■(R_it = 〖γ_Ri+R〗_(it-1)+γ_R I_(it-1) +ε_Rit @D_it = 〖γ_Di+D〗_(it-1)+γ_D I_(it-1) +ε_Dit ))***(2)**

The additional index *i* refers to the state; therefore, *I_it_* represents the number of infected people in state *i* at time *t*. Consistent with the panel data specifications, we added a state-specific “fixed effect” to each of the equations, *γ_i_*, which represents time-invariant state characteristics such as population rate. The *ε_.it_* represent error terms.

We apply the dynamic panel data approach to the number of positive test results per day as reported on internet sites. To avoid imposing too much specificity, we allowed for some flexibility in the functional form by including the number of tests both linearly and quadratically and as a proportion of the population:


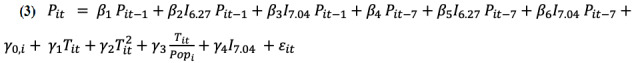


where *P_it_* is the number of new positive test results and *T_it_* is the number of tests administered in state *i* on day *t*; *I_6.27_* and *I_7.04_* are indicator variables for the time periods from June 27-July 3 and July 4-10, 2020, respectively (latest available data at the time of analysis); and *Pop_i_* is the population of state i (assumed to be constant during the sample). Equation 3 is readily interpretable. The terms containing a *β* parameter represent the dynamic component of the model. The first term on the right side represents a day-to-day persistence effect (ie, every new case the previous day is a risk factor that contributes *β_1_* new cases to the current day’s caseload). The next two terms allow for shifts in this risk factor (additions or subtractions) for the weeks beginning June 27 and July 4. Analogously, the next three terms represent a 7-day persistence effect and shifts in that effect for the weeks beginning June 27 and July 4. The 7-day persistence effect is the approximate modal time between viral contraction and the appearance of symptoms; therefore, it is related to the reproduction rate (R parameter) in structural contagion models. The final five terms of Equation 3 contain all the contemporaneous effects in the model (the nonhomogeneous component of the difference equation), as in, all the time subscripts occur contemporaneously at time *t* except for the state fixed effects, which by definition do not change over time. The first of these terms represents state-specific effects, which are an important control variable in the panel models. The next two terms are linear and quadratic terms of the number of tests administered, while the third term is the number of tests per person. The next three terms represent the effects of the number of tests administered. The fourth term allows for a shift or discontinuity in the level of new infections for the week of July 4-10 because of increasing concern that the pandemic has re-emerged, particularly in the previous 7 days. We would associate a positive shift with an underlying increase in infection rates. The final term is an error term that represents all types of measurement errors.

### Data Sources

Case and test data, including the total number of tests administered and the number of positive results, were taken from the COVID Tracking Project [84], which compiles data from multiple sources. Data were accessed from GitHub [85] after 6 PM on July 10, 2020, so that the data would be complete for that day. Population estimates were derived from the 2019 annual state estimates from the US Census Bureau [86].

### Estimation

There are three problems with the specification of Equation 3 for estimation purposes. First, the inclusion of lagged dependent variables on the right side means that the errors are autocorrelated and that the usual exogeneity restrictions are violated; therefore, least squares estimates are inappropriate. Second, some variables are omitted, such as all the variables represented in extensions of the SIR model, and other variables that represent socioeconomic factors influencing the contagion, testing, and reporting processes may also have been omitted. Third, our data set has a relatively short time duration, and the asymptotic properties of fixed-effects or random-effects panel data estimators such as statistical efficiency or normality apply as *t*→∞. Use of these estimators with small values of *t* creates a small-sample problem with unknown or undesirable estimator properties. We applied the Arellano-Bond approach [87,88], which has improved properties for small samples and is appropriate for application to data sets with a small *t* and large *i*.

Fortunately, DPD methods can be used to specifically resolve these statistical problems [89-95]. DPD models allow direct estimation of difference equations with panel data, which resolves multiple problems that appear in the COVID-19 data [96]. The technique we used was developed by Arellano and Bond [87], who applied a generalized method of moments (GMM) approach to a dynamic formulation of employment equations, such as the influence of employment levels in a previous period on employment levels in the current period [97-99]. The basic concept translates to the COVID-19 pandemic in the sense that the number of infections in the current period is a function of lagged infection numbers and other variables. In addition, the DPD removes the individual state effects by first differencing the model. Regressions that include a lagged value of the dependent variable violate the exogeneity restrictions for ordinary least squares and panel estimators such as fixed or random effect models because the lagged dependent variable will be correlated with the error term. DPD model estimation is an application of Hansen’s GMM approach to difference equations estimated from panel data [97,100-102]. The GMM approach solves the endogeneity problem [103,104]. Rather than minimizing a loss function such as the sum of squared errors or maximizing a distribution-specific likelihood function, the GMM approach focuses on the identification of restrictions, including exogeneity restrictions. In an estimable model, there are more identifying restrictions than parameters, and the GMM selects the parameter values that come closest to satisfying the overidentifying restrictions [105]. In our application, we used 10 explanatory variables as defined in Equation 3 and 940 overidentifying restrictions (ie, the same order of magnitude as the sample size n=1040); therefore, the degrees of freedom were more than sufficient for statistical inference. The GMM procedure requires a set of instrumental variables; in the case of DPDs, the instruments include lags and/or lag differences in the Y variables. These instruments help resolve the endogeneity problem as well as the omitted-variables problem. In addition to addressing the theoretical concerns inherent in the estimation of any difference equation model, the DPD approach addresses multiple statistical issues that are likely to occur in COVID-19 data.

First, the GMM approach is asymptotically efficient; however, it also has good small sample properties, including samples with a large cross-section and a small number of time periods [102]. This is especially important for statistical analysis early in pandemics, when data are not available for a long period of time, as well as for our testing of whether changes in the transmission rate (that may have occurred 1 to 2 weeks ago) have affected the number of positive test results in the past week.

Second, this approach is robust to omitted variables because of its reliance on identifying restrictions and instrumental variables. This is important because we estimate a relatively sparse model that does not include direct controls for mediating factors, data collection issues, or reporting idiosyncrasies.

Third, the approach includes statistical testing of the overidentifying restrictions (ie, whether the empirical model and estimation technique are statistically valid). For this test, we used the Sargan chi-square test.

Fourth, this approach corrects for autocorrelation.

A significant drawback to DPD methods is that they are computationally complex and become very time- and resource-intensive as the number of observations grows.

We used the Arellano-Bond estimation technique developed specifically for DPD applications. We implemented the Arellano-Bond technique using the *xtabond* command in Stata 16.1 (StataCorp LLC).

### Model Validation

To validate the significance of the regression, we used a Wald chi-square statistic to test the null hypothesis that the independent variables did not explain the dependent variable (standard goodness-of-fit measures such as *R*^2^ are uninformative in models with a lagged dependent variable). To test the appropriateness of the model, we applied the Sargan chi-square test. This is a test of the null hypothesis that the (over)identifying restrictions of the model are statistically met; heuristically, this null hypothesis means that the model and estimation procedure are valid. We used α≤5% for tests of statistical significance.

### Model Parameters

We report the point estimates and the *P* values for all model parameters in Equation 3 as well as additional statistical test results and *P* values for combinations of parameters when of interest. Of interest are the null hypotheses: *β_2_* = 0, *β_3_* = 0, *β_5_* = 0, and *β_6_* = 0. These hypotheses jointly represent the hypothesis that there has been no change in the persistence of the pandemic (ie, the number of new COVID-19 cases over the past two weeks has remained relatively constant). We interpreted rejection of one or more of the hypotheses as evidence that the pandemic is evolving differently, with positive parameter values associated with greater persistence and a re-emergence of the pandemic.

### Surveillance Reporting

We translated the estimation results into a surveillance reporting context. The dynamic component (Equation 3) is presented in terms of the persistence rate per 100,000 cases, defined as the number of new COVID-19 cases in every 100,000 cases that remained constant, and this component was applied to the reported infection numbers to determine its effect on the number of cases per state per day. The contemporaneous component was applied to the reported infection numbers to determine its effect on the number of cases per state per day. The two effects were added to obtain a modeled total number of cases per state per day, and this number was multiplied by 52 to obtain a national figure (including the District of Columbia and Puerto Rico but excluding other territories).

## Results

### Data

The internet data mining effort resulted in a panel (longitudinal data set) with 52 “panels” (50 states, the District of Columbia, and Puerto Rico) using observations from June 13 through July 10, 2020. Before the analysis, outlying and negative values were crosschecked with other reputable COVID-19 data tracking websites, including USA Facts [106] and the Johns Hopkins Coronavirus Resource Center [107]. The data set has m = 52 × 28 = 1456 observations. Because the model requires 8 days of observations to account for various lags and differencing, the model estimation uses n = 52 × 20 = 1040 observations.

### Estimation Results

We present the estimation results in Table 1.

**Table 1:** Arellano-Bond dynamic panel data modeling of the number of daily infections by state from March 20 to July 10, 2020.

Estimation		Coefficient	*P* value
**Variables**			
	Lagged daily positive cases	0.0630	.31
	Lagged daily positive shift, June 27-July 03	0.0977	.14
	Lagged daily positive shift, July 04-10	–0.1727	.009
	Seven-day lagged daily positive cases	0.5188	<.001
	Seven-day lagged daily positive shift, June 27-July 03	0.0118	.90
	Seven-day lagged daily positive shift, July 04-10	0.2691	.002
	Constant	17.7791	.68
	Daily tests	0.0520	<.001
	Daily tests squared	–1.54 × 10^-7^	.002
	Daily tests / population	–86,527	<.001
**Fitness measurements**			
	Wald test of regression significance (χ^2^_10_)	1489.84	<.001
	Sargan test of overidentifying restrictions (χ^2^_946_)	935.52	.59
	Test of lagged daily positive cases + shift July 04-10 = 0 (χ^2^_1_)	–9.92	.002

### Model Validation

To examine the model fit, we applied a Wald chi-square test of the null hypothesis that there is no explanatory power in the explanatory variables. The model was statistically significant (χ^2^_10_=1489.84, *P*<.001). The Sargan chi-square test failed to reject the null hypothesis of valid overidentifying restrictions (χ^2^_946_=935.52, *P*=.593).

### Model Parameter Estimates

The coefficient on the lagged dependent variable of the number of daily cases that tested positive on the previous day was positive and statistically significant (0.0630, *P*<.001). The shift values for this parameter for the weeks beginning June 27 and July 4, 2020, are 0.0977 (*P*=.138) and –0.1727 (*P*=.009), respectively. The effective parameter value for the week of July 4 is 0.0630 – 0.1727 = –0.1097 (*P*=.002).

The coefficient on the 7-day lagged dependent variable, the number of daily cases that tested positive 7 days earlier, was positive and statistically significant (0.5188, *P<*.001). The shift values for this parameter for the weeks beginning June 27 and July 4, 2020, are 0.0118 (*P*=.897) and 0.2691 (*P*=.002), respectively. The effective parameter value for the week of July 4 is 0.5188 + 0.2691 = 0.7879 (*P*<.001).

The coefficient on the linear term in the number of daily tests administered was positive and statistically significant (0.0520, *P*<.001), and the coefficient on the quadratic term was negative and statistically significant (–1.54e-07, *P*=.002). The coefficient on the number of daily tests per person was negative and statistically significant (–86,527, **P*<.001*).

### Surveillance Results

Table 2 translates the statistical results into a user-friendly, intuitive surveillance reporting template. The first two rows are the reported number of cases and tests, respectively. The third row is the estimated 1-day persistence rate, as in, the number of cases estimated on the current day for every 10,000 cases the previous day. The fourth row is the 7-day persistence rate (ie, the estimated number of cases on the current day for every 10,000 cases 7 days prior). The fifth row is the estimated dynamic component of the model in terms of the number of cases per state per day. This was determined by applying the persistence rates from rows 3 and 4 to the average reported number of cases and adding the effects. The sixth row is the estimated contemporaneous component of the model in terms of number of cases per state per day. The seventh row sums the dynamic and contemporaneous effects to obtain the total estimated effect, as in, the estimated number of new positive test results per state per day. The first column contains the described information as state averages for the period of June 27 to July 03, 2020. The second column contains the information for the United States in aggregate. The third and fourth columns show the same data as the first two columns but for the period of July 4 to 10, 2020.

**Table 2:** Dynamic panel data estimation results for the United States from June 27 to July 10, 2020.

Variable	June 27-July 3		July 4-10	
	State average	National average	State average	National average
Daily average number of cases for the week	909	47,278	1048	54,491
Daily average number of tests for the week	12,281	638,619	12,630	656,741
Estimated daily persistence rate (per 10,000 cases)	1607	1607	–1106	–1106
Estimated 7-day persistence rate (per 10,000 cases)	5306	5306	7816	7816
Estimated dynamic component (number of cases per day)	499	25,968	595	30,923
Estimated contemporaneous component (number of cases per day)	466	24,254	490	25,464
Total number of estimated cases per day	966	50,221	1084	56,387

## Discussion

### Principal Findings

Our primary findings are that the 7-day persistence rate is statistically significant and important in magnitude and that the 7-day persistence rate increased by almost 50% from the week of June 27-July 3 to the week of July 4-10 (Table 1). The increase in the 7-day persistence translates into an increase from 5306 new cases per 10,000 cases 7 days prior to 7816 new cases per 10,000 cases (Table 2). On average, this resulted in 95 new cases per state per day. Coupled with a modest increase in the contemporaneous component, the combined result is an estimated increase of 118 new cases per state per day or 6166 new cases nationally per day. The increase in the number of new cases per day is indicative of a shift in the underlying contagion transmission and corroborative of the statement that reopening the US economy has increased the contagion reproduction rate.

The coefficients on the daily lagged dependent variable are small in magnitude and do not indicate strong day-to-day persistence. The negative estimated daily persistence rate for the week of July 4 is indicative of a daily “snaggle-tooth” pattern in the number of daily cases at the state level. This simply indicates that a low number of cases on one day is offset by a high number of cases the next day, probably due to reporting delays and differential testing periods; this pattern appears slightly in the US aggregate data and is strongly evident in the California data. Other states exhibited different snaggle-tooth patterns, including high-incidence states such as Florida, Texas, and Georgia.

The contemporaneous component of the model contributed positively to the number of new daily cases but did not change significantly over the sample period.

### Limitations

While DPD is useful in deriving dynamic estimates of the rate of transmission of COVID-19, static numbers using traditional surveillance tools must also be included to obtain a complete understanding of the pandemic.

### Conclusions

The DPD model is a statistically validated analysis of reported COVID-19 data and an important addition to the epidemiological toolkit for understanding the progression of the pandemic. It is important to recognize that this is a supplementary tool that does not replace detailed contagion modeling with detailed and specific data for accurate representation of contagion model parameters. However, there are four salient advantages of the DPD approach. First, this approach enables statistically efficient extraction of information from existing data sets, including statistical validation of results; therefore, it is applicable to the most commonly tracked and reported data in the current pandemic. Second, the tool could be applied relatively quickly after the pandemic started because of its ability to model reported data rather than detailed contract tracing data, which is largely unavailable to date. That is, changes in the evolution of the pandemic can be confirmed much more quickly using panel data than using aggregate data. Third, this approach informs real-time policy decisions, including decisions based on commonly reported data, such as reopening state economies. Fourth, the model results can help inform the parameterization of more traditional contagion models.

This model is consistent in that it shows a higher reproduction rate during the most recent 7 days; this confirms that in general, normal operation should not be resumed in the United States. Rather, empirically validated public health guidelines such as wearing masks, social distancing, social isolation, hand washing, and avoidance of social gatherings should be immediately adopted to reduce the contagion. In fact, White House guidelines recommend 14 sustained days of reduced COVID-19–related deaths, new infection cases, and proportions of positive test results prior to reopening. That threshold has not been met. While these findings reflect the national average, it is possible that some areas within the United States meet the White House guidelines, even though reopening is contraindicated in general.

The opening of America involves two certainties. First, the United States will be COVID-19–free only when there is an effective vaccine. While scientists are working at unprecedented speed worldwide to develop a SARS-CoV-2 vaccine [6,108-113], realistically, it will be necessary to rely on best public health practices to minimize COVID-19 infection and mortality for at least one more year [110,114-116]. Second, the “social” end of the pandemic will occur before the “medical” end [117]; therefore, improved surveillance metrics are needed to inform health policy on opening sections of America more safely.

## Abbreviations

DPD: dynamic panel data
GMM: generalized method of moments
R_0_: basic reproduction number
SIR: susceptible-infected-recovered
